# Can Hearing Aids Improve Physical Activity in Adults with Hearing Loss? A Feasibility Study

**DOI:** 10.3390/audiolres15010005

**Published:** 2025-01-18

**Authors:** Maria V. Goodwin, Katelynn Slade, Andrew P. Kingsnorth, Emily Urry, David W. Maidment

**Affiliations:** 1 School of Psychology, Aston University, Birmingham B4 7ET, UK; 2 School of Sport, Exercise and Health Sciences, Loughborough University, Loughborough LE11 3TU, UK; k.slade@lboro.ac.uk (K.S.); a.kingsnorth@lboro.ac.uk (A.P.K.); d.w.maidment@lboro.ac.uk (D.W.M.); 3 Research & Development, Sonova AG, 8712 Stäfa, Switzerland; dr.e.urry@gmail.com

**Keywords:** hearing loss, hearing aids, physical activity, wellbeing

## Abstract

**Background/Objectives**: Adults with hearing loss demonstrate poorer overall health outcomes (e.g., physical health, cognitive functioning and wellbeing) and lower levels of physical activity/function compared to those without hearing loss. Hearing aids have the potential to improve cognitive and wellbeing factors, but there is a dearth of evidence on their impact on physical health outcomes. Evidence on the association between hearing aid provision and physical activity is mostly limited to cross-sectional studies. This research aimed to assess whether a study can be performed to identify whether the provision of hearing aids can improve physical activity. **Methods**: This study employed a preregistered observational (prospective cohort) study design of ten older adults (51–75 years) completed assessments at baseline and again at a six-week follow-up. The participants wore an accelerometer (ActiGraph GT9X) without feedback for the full duration of the study. Feasibility was determined using pre-defined criteria, including study drop-out, adherence to accelerometer use and willingness. A battery of health outcomes was also assessed at baseline and follow-up. **Conclusions**: Overall, this study was perceived favourably, with all participants reporting that they enjoyed taking part. Participant retention was 100%, and adherence to the wrist-worn accelerometers was “good” (70%). However, recruitment was challenging, and some participants found the accelerometers to be burdensome. Descriptive statistics for all outcome measures showed non-significant changes in the expected direction (e.g., improved physical activity, cognition and wellbeing). Although the study was well received by participants, modifications to the recruitment strategy and activity tracking procedures are necessary before future large-scale trials assessing the effectiveness of hearing aids on physical activity can be undertaken.

## 1. Introduction

The World Health Organisation [1] estimates that the number of people experiencing disabling hearing loss will increase from 466 million to over 900 million by 2050 due, in part, to the global ageing population. Hearing loss in older adults is associated with poorer health outcomes across a range of domains (e.g., psychosocial wellbeing, cognitive functioning and physical health) [2,3,4] and lower physical activity levels and physical functioning [5,6]. Hearing aids are currently one of the primary clinical management strategies for hearing loss and have been shown to have the potential to improve cognitive function [7] and social–emotional factors, such as quality of life and loneliness [8,9]. However, there is a paucity of evidence assessing whether hearing aids can improve physical health outcomes.

Epidemiological evidence demonstrates that adults with hearing loss are less likely to meet physical activity guidelines than those without hearing loss [5,9,10]. Notably, evidence from the English Longitudinal Study of Ageing (ELSA) found a more rapid decline in physical activity for older adults with hearing loss compared to those without [10]. Declining physical activity is associated with accelerated ageing and an increased risk of developing chronic diseases [2,5,11]. Therefore, improving physical activity in adults with hearing loss is important. Nevertheless, whether the provision of hearing aids can facilitate physical activity in this population remains unclear.

Current evidence regarding the association between hearing aid provision and physical activity is mixed; some research indicates that hearing aid use removes the association between hearing loss and physical inactivity (i.e., improves physical activity to the level of individuals without hearing loss) [9]. Other cross-sectional studies have found that hearing aid use is not associated with overall increased activity [12,13], although one study found that hearing aid users were more likely to engage in walking activity [12]. Most of the current evidence comes from cross-sectional studies, where causation cannot be inferred. Only one longitudinal study has been conducted, which found that there was no change in physical activity after hearing aid fitting [14]. However, this study relied on self-reported physical activity data, which can suffer from recall issues, hindering the accuracy of activity estimates [15]. Additionally, a recent qualitative study that assessed the barriers and facilitators to physical activity in older adults with hearing loss [16] found that hearing aid use acted as both a facilitator in terms of improved communication and as a barrier due to discomfort and poor cleanliness. On this basis, it is apparent that further evidence is required to assess the impact of hearing aids on physical health outcomes in adults with hearing loss.

In line with the Medical Research Council guidelines for developing complex interventions [17], we planned a feasibility study to assess whether a large-scale trial could be performed. On this basis, the current study aimed to (1) determine the willingness of older adults with hearing loss to take part in the study, as well as retention through to follow-up, where study completion was the primary endpoint, (2) assess the implementation of the study delivery and fidelity and (3) quantitatively measure a battery of health outcomes (physical activity, physical function, cognitive function, wellbeing and cardiovascular health) before and after a hearing aid fitting.

## 2. Materials and Methods

### 2.1. Design and Participants

An observational study design (prospective cohort) was used to recruit adults aged 45–75 years old who were able to stand (aided or unaided). This age range was specified by the Ethics Committee due to the nature of some of the outcome measures employed (i.e., balance, gait speed and sit-to-stand), which may have placed older adults at great risk of falls. Inclusion criteria deviated from the pre-registered protocol (https://osf.io/84wyu/; accessed on 20 April 2023). In addition to individuals who were either first-time hearing-aid users (defined as about to be fitted with their first hearing aid or received their first hearing aid in the last 12 months), the study also invited individuals who already used hearing aids (i.e., existing users) to participate. Deviation occurred due to difficulties with recruitment, where it became apparent that recruiting only first-time hearing aid users was not feasible (i.e., a general unwillingness to take part in research due to hearing loss/aid concerns). Individuals were not eligible to take part in the study if they did not speak fluent English or had a diagnosis of dementia (self- or family-reported) due to not being able to provide informed consent. Recruitment occurred through Boots Hearingcare centres located in the East Midlands (United Kingdom, UK) and local advertisements in community centres located in the town of Loughborough, UK. Participants were reimbursed with GBP £10 Amazon vouchers for each visit that they attended (a total of GBP £20 if both visits were attended). We aimed to recruit 20 participants, with an anticipated drop-out rate of 25%. The observational design meant that the blinding of participants or outcome assessors was not possible. This study was conducted at Loughborough University UK and was approved by the Ethics (Human Participants) Sub-Committee (Ref. 11177).

### 2.2. Outcome Measures

All self-report variables were assessed using validated measures and followed their respective published scoring guidance at both baseline and six-week follow-up unless otherwise stated. Brief explanations have been provided for each measure, but further information can be found in the preregistered protocol (https://osf.io/84wyu/; accessed on 20 April 2023; accessed on 20 April 2023) and the references cited. Two changes to the pre-registered protocol were made. Namely, technical difficulties (system failure and data unretrievable) for two of the planned cognitive assessments—backward digit and spatial span tasks meant that no data were available for these tests.

#### 2.2.1. Primary Outcome Measures

##### Adherence and Implementation

As this was a feasibility study, we assessed the willingness of participants to engage in and complete the study (adherence) and to assess the implementation and fidelity of the study.

***Willingness to take part.*** Study completion at the follow-up session was the primary endpoint, and a retention rate of >80% was used to indicate whether the study could proceed to a full trial.

***Implementation and fidelity.*** Detailed research notes were taken to help inform a potential full-scale trial regarding any difficulties with participant recruitment, and participants reported difficulties with any aspects of the study. To assess the acceptability of the study, a 12-item measure was used (Supplemental Materials, S2). The responses were recorded on a five-point Likert scale from one (*strongly disagree*) to five (*strongly agree*). This measure was developed in line with the Theoretical Framework of Acceptability [18] and demonstrated good internal consistency (Cronbach’s Alpha = 0.70). Furthermore, participant fidelity (wearing of the activity monitor) was determined based on time spent wearing the device during the study period. Average weekly data were calculated if the participants had worn the device for at least 72 h (3 days) over a seven-day period for the duration of the study. We used a traffic light system to evaluate this, where weekly available accelerometer data for >80% of the study period were classified as ‘green’ (study can proceed to full trial), data for 40–80% as ‘amber’ (study can proceed but the monitoring of physical activity requires adjustments) and data <40% as ‘red’ (proposed method of monitoring physical activity not feasible and should not be included in a full trial).

#### 2.2.2. Secondary Outcome Measures

##### Physical Activity

***Accelerometer measured physical activity.*** At the baseline assessment, participants were provided with an ActiGraph GT9X accelerometer (ActiGraph, USA) to be worn on the non-dominant wrist. The devices were set to 100 Hz per minute with a blank screen to reduce influencing participant behaviour during the study period and to prolong battery life. Participants were shown how to use this during the baseline assessment session and asked to wear it throughout the study (i.e., until they attended the six-week follow-up session). Data were processed and analysed using the GGIR 3.0 R-package [19]. Daily Moderate–Vigorous Physical Activity (MVPA) was calculated using the metabolic equivalent (MET) rates. Following guidance from previous research [5,20,21], MVPA was only calculated for individuals with data of at least 72 h (3 days) of device wearing.

***Self-report physical activity.*** This was assessed using the International Physical Activity Questionnaire (IPAQ [22]). The total weekly MVPA was calculated following the IPAQ guidelines.

##### Physical Function

***Short Physical Performance Battery (SPPB)*** [23]. This validated measure of physical function classifies performance into three domains: balance, gait speed and sit-to-stand. The scores range from 0 to 12 and are classified as no/minimal limitations (10–12), mild limitations (7–9), moderate limitations (4–6) or severe limitations (0–3).

##### Cardiovascular Health

Participants reported any diagnosis of diabetes (type 1 or 2), stroke, hypertension or any other cardiovascular condition. Each condition was scored as a binary response (0 = no, 1 = yes). In addition, a CardioChek Point of Care Bio Analyser was used to conduct capillary blood sampling. Readings were taken in real time directly from the machine. Cholesterol was assessed with total, High-Density (HDL) (mmol/L) and the total/HDL ratio. Glucose was measured with HbA1c (%). Resting heart rate and blood pressure were also recorded.

##### Cognitive Function

***Montreal Cognitive Assessment–Hearing (MoCA-H)*** [24]. This dementia screening tool was specifically designed and validated for individuals with hearing loss. It provides a general measure of overall cognitive function. The measure provides a score from 0 to 30, with a score of <24 indicative of cognitive decline.

***Digit and Spatial Span Tests.*** EPrime v3 software was used to visually deliver the forward and backward digit-span and spatial-span (Corsi-block) tests. The standard EPrime tests were used in line with WAIS-III [25] and total scores (maximum number of digits/blocks remembered) were recorded.

##### Loneliness

***UCLA Loneliness Scale*** [26]. This 20-item scale is responded to on a scale from ‘*never*’ (1) to ‘*always*’ (4), producing a continuous score from 20 to 80, where a higher score indicates greater loneliness.

##### Depression

***Hospital Anxiety and Depression Scale (HADS)*** [27]. This 7-item sub-scale allows participants to respond to questions on a scale from 0 to 3, creating a continuous score of 0–21, where a higher score indicates greater symptoms of depression.

##### Social Isolation

***The Lubben Social Network Scale–6*** [28]. Participants respond to six statements about social contacts (three for family and three for friends). These responses create a continuous score from 0 to 30, where a lower score indicates greater social isolation.

##### Wellbeing

***The World Health Organisation (WHO)–5 Wellbeing Index*** [29]. Participants respond to five statements, which are scored from ‘*at no time*’ (0) to ‘*all of the time*’ (5). This creates a total continuous score of 0–25 that is multiplied by four to create a percentage, where a higher score represents greater wellbeing.

***The Short Warwick–Edinburgh Mental Wellbeing Scale*** [30]. This scale assesses mental wellbeing and consists of seven items scored from ‘*none of the time*’ (1) to ‘*all of the time*’ (5). This creates a total continuous score of 7–35, where higher scores represent better mental wellbeing.

##### Cognitive Fatigue

***Vanderbilt Fatigue Scale*** [31]. This scale provides an overall measure of fatigue, as well as domain-specific subscales (emotional, social, physical and cognitive). Responses are coded from ‘*never/almost never*’ and ‘*strongly disagree*’ (0) to ‘*almost always/always*’ and ‘*strongly agree*’ (4). This gives a continuous scale from 0 to 160 (each subscale 0–40), where higher scores indicate greater fatigue.

#### 2.2.3. Other Measures

##### Demographics

The following demographic information was recorded during the baseline session only: age (years), gender identity, ethnicity, highest level of education obtained, years hearing problems have been experienced and type of hearing aid.

##### Hearing Aid Outcomes

The International Outcome Inventory for Hearing Aids (IOI-H [32]) is a seven-item scale assessing hearing aid use, benefit, residual activity limitations, satisfaction, residual participation restrictions, importance to others and quality of life. Responses for each item are coded on a scale from 1 to 5, resulting in a continuous scale from 7 to 35. Higher scores indicate greater hearing aid outcomes. The IOI-HA was assessed during the six-week follow-up session only.

##### Hearing Status

Provided during the baseline session only, audiograms were provided from the participant’s most recent hearing assessment, enabling hearing status to be categorised following the WHO guidelines [33], whereby the grade of hearing in the better hearing ear was classified as either mild (20–34.9 dB HL), moderate (35–49.9 dB HL), moderately severe (50–64.9 dB HL) or severe (65–79.9 dB HL).

### 2.3. Procedure

A more detailed description of the study procedure is provided in the pre-registered protocol (https://osf.io/84wyu/). Participants were screened for eligibility by clinic-based audiologists at Boots Hearingcare and provided with a study information sheet. Individuals expressing an interest in taking part in the study then emailed the study researcher (MVG), who arranged to meet with them online via MS Teams. During this online meeting, MVG verbally outlined the study, screened for eligibility and allowed potential participants to make an informed decision as to whether they wished to participate. We aimed for baseline assessments to be approximately one week before the hearing aid fitting, with follow-up approximately six weeks after. In addition, prior to attending the in-person baseline session, audiologists provided the participants with a copy of their most recent audiogram.

#### 2.3.1. Baseline Assessment

On arrival, participants completed a consent form, followed by a standardised Health Screening Questionnaire (Supplemental Materials, S1) and demographic questions. Following this, participants were asked to sit for five minutes to allow for resting heart rate and blood pressure to be recorded. Participants then completed the three cognitive assessments in a randomised order. MoCA-H was delivered using flash cards as per MoCA-H guidelines. The digit- and spatial-span tests were administered on a laptop. The SPPB was then delivered by the researcher. After this, the IPAQ, UCLA Loneliness Scale, HADS, Lubben Social Network Scale, WHO-5, Short Warwick–Edinburgh Mental Wellbeing Scale and Vanderbilt Fatigue Scale were completed. Participants then had their cholesterol and glucose levels checked using the CardioChek Point of Care Bio Analyser. Lastly, participants were provided with the ActiGraph GT9X device, with the devices set to a blank screen. The participants were provided with a charger and asked to charge the accelerometer overnight once a week. Text reminders were sent to participants on these days with a request to confirm the device had been sufficiently charged. Participants were also given an instruction booklet (Supplemental Materials, S3) that included information on how to use and charge the accelerometer, as well as providing space to record when they charged the device (time removed and time put back on), any issues they had with the device, days they forgot to wear it or any other information they felt was relevant (e.g., any issues experienced).

#### 2.3.2. Follow-Up Assessment

The follow-up assessment occurred approximately six weeks after the baseline assessment; several participants completed the baseline assessment less than seven days before their hearing aid fitting (first-time users only), which led to a slightly shorter study duration than anticipated. The follow-up assessment followed the same format except that, after completing the informed consent forms and Health Screening Questionnaire, the accelerometer device was removed and returned to the researcher, along with the booklet. In addition to the self-reported measures in the baseline assessment, hearing aid outcomes and use were assessed using the IOI-H, and participants completed the acceptability measure (Supplemental Materials, S2).

### 2.4. Data Analysis

As this was a feasibility study, it was not sufficiently powered to test for statistical differences between baseline and follow-up outcomes. However, mean scores (and standard deviations) were compared between the baseline and follow-up sessions for each outcome measured. Repeated measure (related samples) *t*-tests were used for continuous outcome data, and Wilcoxon signed rank tests for ordinal or non-normal data. Descriptive statistics were used to assess the acceptability of the study.

## 3. Results

**Figure 1:**
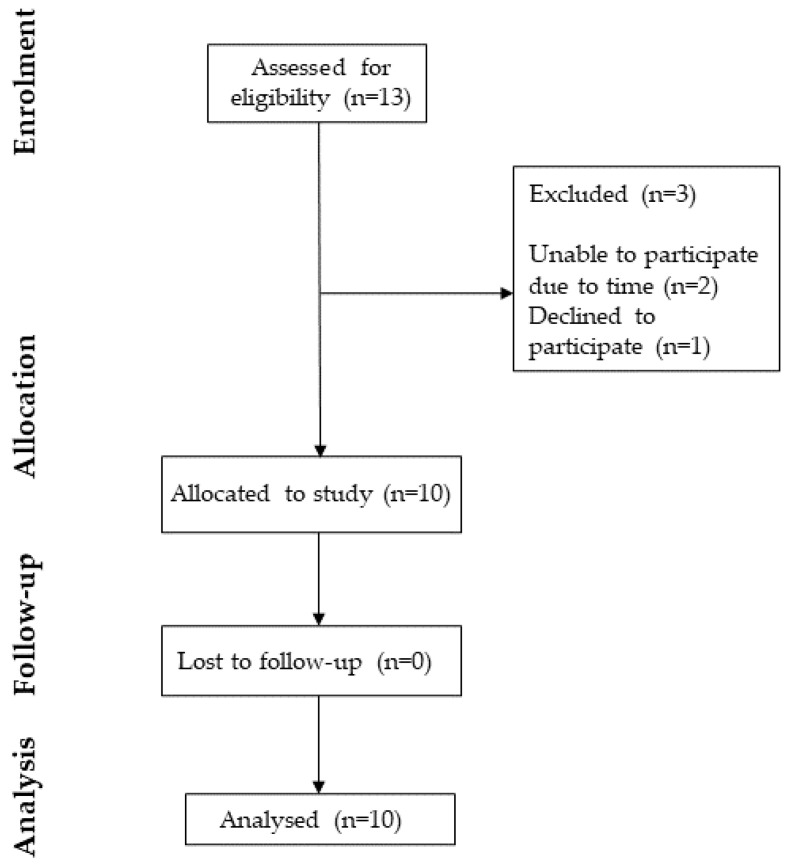
Adapted CONSORT flow diagram summarising the steps and number of participants excluded at each stage, culminating in the final number for analysis.

Recruitment for the feasibility study began in February 2023 and continued until August 2023; all follow-up assessments were completed by September 2023. Thirteen individuals initially expressed interest in participating in the study, two were unable to participate due to time commitments and one declined to participate after initial discussion. Ten participants completed the baseline assessments, and all participants were retained for follow-up (Figure 1). The time between baseline and follow-up was approximately six weeks (*M* = 5.93; *SD* = 0.43 weeks).

The final sample consisted of ten adults aged 51–75 years (*M* = 65.9 years; *SD* = 8.1). Most were male (60%), white British (90%) and had at least a bachelor’s university degree (60%). Half of the participants were new hearing aid users; most used behind-the-ear (BTE) hearing aids (80%) and had mild to moderate hearing loss (60%). Full baseline demographics are presented in Table 1.

### 3.1. Primary Outcome Measures

#### Adherence and Implementation

The retention rate for the study was 100%, which met our criteria for the progression to a full-scale trial. However, we were unable to reach the planned sample size of 20 participants despite expanding the eligibility criteria to include both first-time and existing hearing aid users.

For the wrist-worn activity trackers, complete activity data for six weeks (≥72 h per week) were available for seven participants (70% of the sample). Data from the other participants were unavailable due to problems with the hardware (i.e., unable to connect ActiGraph to computer; *n* = 1) and software (i.e., data unretrievable; *n* = 2).

**Figure 2:**
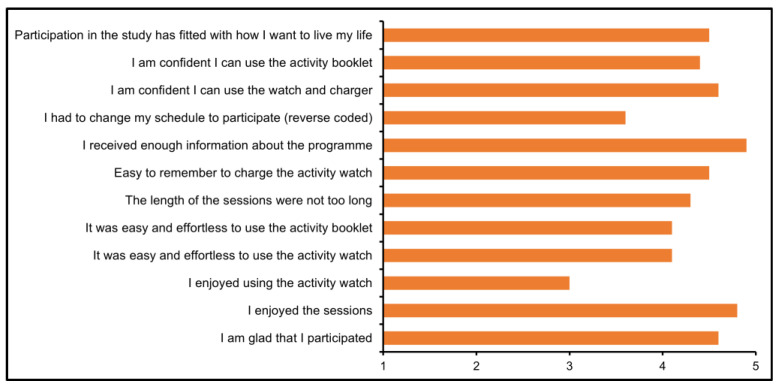
Participants reported acceptability of the study experience (mean scores for each item). Values represent Likert scale responses from 1 (strongly disagree) to 5 (strongly agree).

Overall, participants perceived the programme favourably (Figure 2), with all reporting that they enjoyed taking part in the study. One participant stated, “*I had fun doing it [the laboratory sessions], I didn’t find it excessive or strenuous*”. Most felt confident using the wrist-worn accelerometer and charger (90%) and the activity booklet (80%). Regarding tracking physical activity, some (30%) stated that they did not enjoy using the accelerometer and found the accelerometer to be “*annoying*” to wear all the time. However, most found the watches easy to use (80%) and remembered to regularly charge the device (90%). Some participants also commented that they would prefer fewer activities using the computer (i.e., the cognitive tasks) during the in-person sessions.

### 3.2. Secondary Outcome Measures

Table 2 presents the differences for each outcome measure from baseline to the six-week follow-up; none demonstrated a statistically significant difference at follow-up (*p* ≥ 0.059), although most showed changes in the expected direction (i.e., improved physical activity, physical function, cognitive function, psychosocial wellbeing and cardiovascular health). At follow-up, the participants’ responses to the IOI-H indicated high overall hearing aid outcomes (*M* = 31.90; *SD* = 1.79).

## 4. Discussion

A key aim of this study was to assess the feasibility of a larger randomised controlled trial (RCT) using our pre-registered traffic light system. The study procedure itself was generally well received by participants, but the findings were mixed regarding ‘willingness to take part’. Whilst the retention in the study was classified as ‘green’ (100% retention), there are some concerns over participant recruitment. The necessity for an alteration to the inclusion criteria to recruit both first-time and existing hearing aid users is a clear indicator that, without major changes to the recruitment strategy, a full-scale trial would not be feasible. In addition, the length of time (six months) required to recruit ten participants for the current study raises concerns about the feasibility of a future trial.

There were several explanations for the difficulties we experienced when recruiting first-time users. We were conscious from the outset that this may be a potentially difficult population to recruit from, and this was part of the motivation for conducting a feasibility study. Appointments for first-time hearing aid users can be lengthy (upwards of an hour) and involve a lot of new information, as well as the potential psychological impact of processing the need to manage hearing aids. As such, participants may have felt overwhelmed by the information, so involvement in a research study may have been of less importance. The study also predominantly relied on audiologists recommending the study to their patients/clients, which we found had additional challenges such as competing interests and motivation (spending time discussing research compared to commercial focus), as well as a potentially limited understanding of the importance of research.

Additional considerations regarding the recruitment strategy are necessary before moving forward with a larger study. For example, in this study, recruitment was limited to adults being assessed for and receiving hearing aids from Boots Hearingcare, an independent sector provider of hearing services. Future work should seek to engage both public (i.e., publicly funded UK National Health Service [NHS]) as well as independent sector (e.g., Boots Hearingcare) hearing aid users. This would not only aid recruitment by including a larger pool of potential participants but also have the potential to engage a more representative sample population. Ensuring the inclusion of NHS hearing aid users in future work is critical because hearing loss is known to disproportionately affect those from areas of social deprivation and lower socioeconomic backgrounds [34,35,36].

A further consideration for future work is that data from the accelerometers were missing from 30% of the participants, which suggests that larger trials need to account for both potential drop-out and loss of accelerometer data. Future trials could consider using multiple methods to track physical activity to protect against data loss; for example, previous research with older adults has incorporated both accelerometer data and data from commercial activity watches, as well as self-reported physical activity [37]. That said, of those who had data available, all participants wore the watch for at least 72 h (3 days) for each of the required six weeks, which met the criteria (80%) for proceeding to full trial. This was a novel approach in this area, as most studies track physical activity for one week at baseline and (where applicable) for one follow-up week [5,12,13]. We opted to track activity for the full six weeks to avoid potential changes in behaviour during the monitored week(s) that could occur simply due to wearing the watches. However, this resulted in a greater burden on the participants throughout the study, which was demonstrated by participants’ feedback describing the watches as ‘*annoying*’, especially during the night. A potential future avenue to mitigate the onus on participants whilst maintaining robust measures of activity would be to provide hearing aids with the capacity to track activity. This is a relatively new feature and may be more suitable for individuals who have received hearing aids from the independent sector. However, smartphone-connected hearing aids are also available via the NHS (e.g., Danalogic ‘*Ambio*’), so this may be a viable option in the near future. Recent research demonstrated that this method has the potential for improved accuracy of activity tracking compared to wrist-worn devices [38]. Tracking activity directly through hearing aids would also allow for hearing aid use to be measured objectively (i.e., datalogging). Although the current study measured self-reported hearing aid use using the IOI-HA [32], this only provided a snapshot of self-reported use; where possible, future work should incorporate data from the hearing aid devices for the duration of the study.

Several participants reported that they felt they had to change their schedule to participate in the study (Figure 2), which could have a negative impact on recruitment and retention in future trials if involvement in the trial is perceived to be too burdensome, then potential participants will be less likely to enrol. Indeed, this may account for the recruitment difficulties in the current trial. Although not possible in the present study, future trials could consider onsite assessments (i.e., at the location of hearing assessment) to reduce the impact of travel on schedule changes.

Additionally, the study did not demonstrate any statistically significant differences between baseline and follow-up for any of the secondary outcome measures that were assessed. This was anticipated, as the study was not powered to test for statistically significant differences. The short time frame between baseline and follow-up may also explain why statistical changes were not identified, and future work should include additional follow-up assessments (e.g., at six and twelve months). The inclusion of additional follow-up measurements would also enable us to assess the longer-term impact of hearing aid provision. In addition, the inclusion of existing hearing aid users meant that half of the participants were already using hearing aids at the baseline assessment. Nevertheless, all outcomes changed from baseline to follow-up in the expected direction and could be used to inform a sample size calculation for a future trial. Furthermore, existing evidence on the impact of hearing aids on physical activity also demonstrates that hearing aid provision alone is not associated with an increase in physical activity [14]. Similarly, evidence from qualitative research found that hearing aids were both a barrier and facilitator of physical activity engagement [16]. Collectively, this suggests that hearing aids alone may not be sufficient to improve physical activity in this population, and any further studies in this area should consider incorporating additional behaviour change intervention strategies (e.g., goal setting, activity monitoring, etc.) [16]. Therefore, future research, such as the development of an RCT, should test this further by including additional behaviour change techniques as an intervention group and hearing aid provision alone as an active control.

## 5. Conclusions

Overall, the current results suggest that a full-scale RCT assessing the provision of hearing aids on physical activity in adults with hearing loss would be possible (e.g., high retention and adherence) but with some modifications. Specifically, recruitment strategies to include public healthcare users, revisions to either the product used (hearing aids instead of watches) or length of time to track activity would reduce the burden on participants. With these modifications, a full-scale RCT could provide valuable insights into the role of hearing aids in increasing physical activity, as well as potentially reducing the risk of developing associated chronic health conditions among adults with hearing loss.

## Figures and Tables

**Table 1:** Baseline demographic characteristics of the total sample.

Characteristics	N (%)
Age (years) ^†^	65.9 (8.1)
Gender	
*Male*	6 (60)
*Female*	4 (40)
Ethnicity	
*White British*	9 (90)
*White (other)*	1 (10)
Education	
*Bachelor’s degree*	6 (60)
*No bachelor’s degree*	4 (40)
Pure Tone Average (Better Hearing Ear) ^†^	49.0 (14.0)

Note: ^†^ = values represent mean and standard deviation.

**Table 2:** Baseline and follow-up (6–7 weeks) comparisons for each variable; data represent paired-sample *t*-tests unless otherwise stated.

Variable	Mean (SD)		*t*-Value	*p*-Value
	Baseline	Follow-Up		
Physical Activity				
*Actigraph Sedentary ^‡^* *(mins)*	717.66 (65.80)	729.19 (125.88)	−0.36	.732
*Actigraph Light Activity ^‡^* *(mins)*	183.54 (39.79)	172.57 (57.94)	0.64	.544
*Actigraph MVPA ^‡^* *(mins)*	105.40 (53.63)	109.50 (72.01)	−0.35	.740
*IPAQ Total MET*	4363.75 (2051.99)	4972.56 (3622.92)	−0.85	.419
*IPAQ MVPA MET*	2487.70 (1494.79)	2268.75 (1995.01)	−0.52	.616
Physical Health				
*Physical Function (SPPB total* ^†^*)*	11.50 (2.00)	12.00 (1.25)	−1.89	.059
*Resting Heart Rate*	70.55 (8.56)	68.90 (10.42)	0.71	.498
*Cholesterol (total)*	4.92 (1.65)	4.22 (1.28)	0.21	.846
*Cholesterol (HDL)*	1.50 (0.61)	1.34 (0.21)	−0.95	.398
*Cholesterol (ratio)*	3.33 (0.40)	3.06 (1.04)	1.58	.254
*Glucose (%)*	5.44 (2.51)	4.99 (0.29)	−0.36	.731
Cognitive Function				
*MoCA total*	27.50 (2.01)	27.6 (1.07)	−0.18	.864
*Forward digit span*	5.30 (1.42)	5.80 (1.75)	−0.83	.427
*Forward spatial span* ^†^	6.00 (1.00)	5.00 (1.00)	−0.45	.655
Psychosocial Wellbeing				
*Depression (HADS sub-total)*	6.60 (4.99)	4.70 (4.00)	1.56	.152
*Loneliness (UCLA total)*	35.30 (10.49)	32.80 (10.88)	2.14	.061
*Social Isolation (Lubben Social Network)*	18.40 (4.35)	18.30 (4.83)	0.12	.907
*Wellbeing (WHO-5)*	82.40 (13.09)	79.20 (17.77)	−0.87	.405
*Mental Wellbeing (Warwick-Edinburgh)*	23.30 (12.86)	30.00 (3.797)	−1.58	.148
*Fatigue (Vanderbilt Fatigue Scale)*	40.90 (16.89)	30.60 (22.96)	1.49	.171
Hearing aid outcomes (IOI-HA *)	30.71 (3.15)	31.90 (1.79)	−0.55	.604

Note: ^‡^ = mean minutes per day; ^†^ = represents median and interquartile values; statistical test used is Wilcoxon Signed-Ranks; * *n* = 5 first-time hearing aid users.

## Data Availability

The raw data supporting the conclusions of this article will be made available by the authors upon request.

## Supplementary Materials

The following supporting information can be downloaded at https://www.mdpi.com/article/10.3390/audiolres15010005/s1. S1: Health Screen Questionnaire for study volunteers; S2: Acceptability scale used in the pilot trial; S3: Sample of instruction booklet and diary provided to participants.
