# Supplementary material for: Can Hearing Aids Improve Physical Activity in Adults with Hearing Loss? A Feasibility Study
Source: Audiol Res. 2025 Jan 18;15(1):5. doi: 10.3390/audiolres15010005 (PMC11755568; doi:10.3390/audiolres15010005)
Supplement: Supplementary file 1 [file audiolres-15-00005-s001.zip › audiolres-3372893-supplementary.pdf]

**SUPPLEMENTARY MATERIALS**

***S1. Health Screen Questionnaire for Study Volunteers***

As a volunteer participating in a research study, it is important that you are currently in good health and have had no significant medical problems in the past. This is (i) to ensure your own continuing well-being and (ii) to avoid the possibility of individual health issues confounding study outcomes.

If you have a blood-borne virus, or think that you may have one, please do not take part in this research.

Please complete this brief questionnaire to confirm your fitness to participate:

1. At present, do you have any health problem for which you are:

| | | | | | |
|---|---|---|---|---|---|
| (a) | on medication, prescribed or otherwise | Yes | ☐ | No | ☐ |
| (b) | attending your general practitioner | Yes | ☐ | No | ☐ |
| (c) | on a hospital waiting list | Yes | ☐ | No | ☐ |

2. In the past two years, have you had any illness or injury which required you to:

| | | | | | |
|---|---|---|---|---|---|
| (a) | consult your GP | Yes | ☐ | No | ☐ |
| (b) | attend a hospital outpatient department | Yes | ☐ | No | ☐ |
| (c) | be admitted to hospital | Yes | ☐ | No | ☐ |

3. Have you ever had any of the following:

| | | | | | |
|---|---|---|---|---|---|
| (a) | Convulsions/epilepsy | Yes | ☐ | No | ☐ |
| (b) | Asthma | Yes | ☐ | No | ☐ |
| (c) | Eczema | Yes | ☐ | No | ☐ |
| (d) | Diabetes | Yes | ☐ | No | ☐ |
| (e) | A blood disorder | Yes | ☐ | No | ☐ |
| (f) | Head injury | Yes | ☐ | No | ☐ |
| (g) | Digestive problems | Yes | ☐ | No | ☐ |
| (h) | Heart problems/chest pains……………… | Yes | ☐ | No | ☐ |
| (i) | Problems with muscles, bones, or joints | Yes | ☐ | No | ☐ |
| (j) | Disturbance of balance/coordination | Yes | ☐ | No | ☐ |

| | | | |
|---|---|---|---|
| (k) | Numbness in hands or feet | Yes ☐ | No ☐ |
| (l) | Disturbance of vision | Yes ☐ | No ☐ |
| (m) | Thyroid problems | Yes ☐ | No ☐ |
| (n) | Kidney or liver problems | Yes ☐ | No ☐ |
| (o) | Problems with blood pressure | Yes ☐ | No ☐ |

If YES to any question, please describe briefly if you wish (eg to confirm problem was/is short-lived, insignificant or well controlled.)

.....................................................................................................................................................

Smoking, physical activity and family history

| | | | |
|---|---|---|---|
| (a) | Are you a current or recent (within the last six months) smoker? | Yes ☐ | No ☐ |
| (b) | Are you physically active (30 minutes of moderate intensity, physical activity on at least 3 days each week for at least 3 months)? | Yes ☐ | No ☐ |
| (c) | Has any, otherwise healthy, member of your family under the age of 35 died suddenly during or soon after exercise? | Yes ☐ | No ☐ |

Allergy Information

| | | | |
|---|---|---|---|
| (a) | Are you allergic to plasters? | Yes ☐ | No ☐ |
| (b) | Are you allergic to latex? | Yes ☐ | No ☐ |

If YES to any of the above, please provide additional information on the allergy

……………………………………………………………………………………………………………………………….

Additional questions for female participants

| | | | |
|---|---|---|---|
| (a) | Are your periods normal/regular? | Yes ☐ | No ☐ |
| (b) | Are you on hormonal contraception | Yes ☐ | No ☐ |
| (c) | Could you be pregnant? | Yes ☐ | No ☐ |

(d) Are you taking hormone replacement therapy (HRT)? Yes ☐ No ☐

Are you currently involved in any other research studies at the University or elsewhere?

Yes ☐ No ☐

If yes, please provide details.

………………………………………………………………………………………………………………

Have you recently given blood or been involved with research involving blood samples?

Yes ☐ No ☐

If yes, please provide details.

………………………………………………………………………………………………………………

Please provide contact details of a suitable person for us to contact in the event of any incident or emergency.

Name …………………………………………………………………………………………………….

Telephone Number …………………………………………………………………………………….

Work Home Mobile

Relationship to Participant ……………………………………………………………………...........

Please enter your height in metres (m) and weight in kilograms (kg).

Height (M) …………………………………..

Weight (Kg)…………………………………

***S2. Acceptability scale used in pilot trial.***

Hearing Aid Provision and Physical Activity

For the next set of questions, please indicate (tick or cross) to what extent you agree with each of the following statements.

| | Strongly Disagree | Disagree | Neither Agree nor Disagree | Agree | Strongly Agree |
|---|---|---|---|---|---|
| I am glad that I was asked to participate in this programme. | | | | | |
| I have enjoyed attending the sessions at Loughborough University | | | | | |
| I have enjoyed using the activity tracker. | | | | | |
| It has been easy and effortless to use the activity watch. | | | | | |
| It has been easy and effortless to use the activity booklet. | | | | | |
| The length of the sessions at Loughborough University were not too long. | | | | | |
| It has been easy to remember to charge the activity watch. | | | | | |
| I feel that I have received enough information about the programme. | | | | | |
| I have changed my schedule to participate in the programme. | | | | | |
| I am confident I can use the activity watch and charger. | | | | | |
| I am confident I can use the activity booklet. | | | | | |
| The participation in this programme has fitted well with how I want to live my life. | | | | | |

Please answer the following questions:

1. What was the worst aspect(s) of your experience with study?
2. What was the most useful aspect of your participation in the study?
3. What would you change to make the study more interesting, enjoyable or engaging?
4. Please add any further comments you would like to tell us.

*S3. Sample of instruction booklet and diary provided to participants.*

Loughborough University

## MY ACTIVITY TRACKER

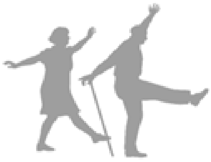

| | |
|---|---|
| **ID NUMBER** | |
| **DATE RECEIVED** | |
| **DATE OF MY HEARING AID FITTING** | |

**IF LOST, PLEASE RETURN TO:**
SCHOOL OF SPORT, EXERCISE & HEALTH SCIENCES
LOUGHBOROUGH UNIVERSITY,
LE11 3TU

**Or contact:** [redacted]

Loughborough University

## HOW TO USE MY BOOKLET

**What is my booklet for?**

This booklet is to help you keep track of how you have used your wrist monitor.

You should only take it off for charging the battery on days 7, 14, 21, 28, 35, and 42, this booklet can help you to track the times you took it off, and any other problems that you may have had.

In this booklet you will also find information on how to wear and how to charge your wrist monitor.

**What should I make a note of?**

You will find that there is space to make notes for every day of the study. You are not expected to make a note on every day.

Please make a note of:

- Any time you take the monitor off and when you put it back on (exact digital time if possible). This includes the day and time that you charged your monitor.
- Any problems you had with the monitor.
- Any days that you did not wear your hearing aids.
- Any problems that you had with your hearing aids.
- Any other comments that are helpful to you.

Loughborough University

## HOW TO USE MY WRIST MONITOR (ActiGraph)

**How do I wear the monitor?**

Wear the monitor every day for the duration the study team have requested. This will be for 7 weeks (1 week before and for 6 weeks after your hearing aid fitting).

Wear the monitor continuously (24 hours/7 days a week).

The monitor must be worn on your <u>non-dominant</u> hand. For example, if you write with your right hand, please place the wrist monitor on your left hand.

Please make sure the monitor is placed on the wrist with the ActiGraph logo <u>at the bottom of the watch</u> (see below).

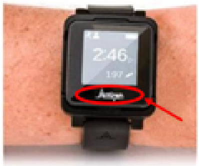


Please make sure that the monitor is snug and secure, like you would wear a watch.

If you experience discomfort on the non-dominant wrist (i.e., sweating under the wrist band, or slight irritation),

Loughborough University

you can place the monitor on the other wrist. <u>Please make sure the orientation is correct, i.e., the ActiGraph logo at the bottom of the watch.</u>

The wrist monitor is waterproof up to 1 meter for 30 minutes, so you can wear it in the shower, bath or for swimming so you should not need to remove it unless specific circumstances require you to do so.

**Note:** The monitor is set to record during set dates i.e., from the day you receive the monitor until the end of the study. Please ensure you wear the device as soon as you receive it. If you have any questions or difficulties with the monitor, please contact the Research Team on [redacted]

**How do I charge the monitor?**

On days 7, 14, 21, 28, 35, and 42 you will receive a reminder from the research team to charge your monitor. Please charge this overnight, (i.e., remove the monitor just before going to sleep, and replace it as soon as you wake up).

1. Remove the monitor from the watchband, firmly grasp and pull up on the device with one hand while

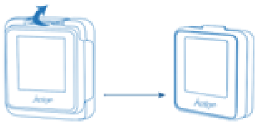

gently lifting the plastic tab on the top edge of the watchband with the other hand (as shown below).

2. Connect the charging dock (see below) to the computer or a wall outlet using the mini-USB cable.

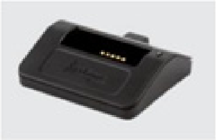

3. Plug the monitor into the dock with the ActiGraph logo facing up. Once connected, the red light on the right side of the dock will turn yellow (see below).

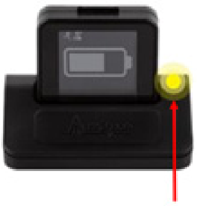

4. Once the device is fully charged, the yellow light will turn green.



5. To replace the monitor, insert it into the wrist strap by positioning the plastic notch on the bottom edge of the device into the matching groove in the bottom edge of the watchband. Make sure that the ActiGraph logo on the device and the 'A' on the watchband are facing in the same direction. Gently push down on the upper portion of the device until it snaps into place.

6. Place the wrist monitor back onto non-dominant hand, following the instructions in '*how do I wear the monitor*'.

**Note.** If you have any difficulties with charging your monitor, please contact the research team on:

[redacted]



**DAY 1 (Day monitor received)**

Date: Nothing to report: ☐

**DAY 2**

Date: Nothing to report: ☐

**DAY 3**

Date: Nothing to report: ☐



**DAY 4**

Date: Nothing to report: ☐

**DAY 5**

Date: Nothing to report: ☐

**DAY 6**

Date: Nothing to report: ☐
